# Employment Responses to a Partner’s Disability Onset (“Care Shocks”): Do Working Conditions Matter?

**DOI:** 10.1093/geronb/gbae208

**Published:** 2024-12-27

**Authors:** Constance Beaufils, Ben Baumberg Geiger, Karen Glaser

**Affiliations:** Department of Global Health & Social Medicine, King’s College London, London UK; ESRC Centre for Society and Mental Health, King’s College London, London, UK; Department of Global Health & Social Medicine, King’s College London, London UK; ESRC Centre for Society and Mental Health, King’s College London, London, UK; Department of Global Health & Social Medicine, King’s College London, London UK; (Social Sciences Section)

**Keywords:** Disability, Employment transitions, England, Spousal caregiving, Working conditions

## Abstract

**Objectives:**

This study examines employment responses to a partner’s disability onset and how this is moderated by working conditions: job satisfaction and psychosocial job demands.

**Methods:**

We use longitudinal nationally representative data from the English Longitudinal Study of Aging. Following the health shock literature, we identify individuals whose partners report the onset of difficulties in activities of daily living (ADL) or instrumental activities of daily living (IADL) between 2 waves (*n* = 1,020) as experiencing a “care shock.” We combine coarsened exact matching and entropy balancing, and logistic modeling to estimate the impact of such a “care shock” on the probability of leaving paid work, working part-time, changing jobs, or looking for a new job. We also explore the moderating effect of gender and working conditions (i.e., job demands and job satisfaction) on the impact of a “care shock” on work transitions.

**Results:**

Our findings show that “care shocks” significantly increase individuals’ likelihood of leaving paid work. This effect is moderated by job demands and job satisfaction. Individuals who report high job demands and job dissatisfaction before the care shock are significantly more likely to leave paid work. In contrast, those with low job demands or job satisfaction show no significant difference in their likelihood of leaving paid work.

**Discussion:**

Our study highlights the role of working conditions in moderating the impact of care shocks on paid work. It informs workplace policies, as our results suggest that adapting working conditions may facilitate participation in the labor market in late career stages.

## Introduction

The UK economy, along with many European countries, increasingly depends on older workers (i.e., aged 50–69), comprising nearly one-third of the UK workforce (Crawford et al., 2021). However, these individuals also play a crucial role in meeting the growing demand for family caregiving, driven by transformations in family life due to sustained increases in longevity and declining fertility (Murphy, 2011; Zigante et al., 2021). For example, in Britain, one in four older female workers has caregiving responsibilities for someone frail or in poor health (Office for National Statistics, 2020). With policies aimed at extending working lives and the noticeable declines in public expenditure on social care for older individuals (Glasby et al., 2021), older workers face increasing pressure to balance work with rising family responsibilities. Therefore, supporting older workers with caregiving duties to remain economically active is critical. An open question is: how can employers and governments effectively assist older workers in navigating work while addressing the mounting demands of family care?

Many studies have focused on the impact of elderly care on older adults’ employment. They highlight the pivotal role of caregiving intensity, with evidence indicating that caregiving negatively affects paid employment and positively affects early retirement for co-resident caregivers or those with long care hours (Lilly et al., 2007; Moussa, 2019). Yet, there remain major gaps in our knowledge about how older people respond to care constraints. First, most prior research has not considered how people can accommodate these constraints at work. In particular, how working conditions moderate an individual’s ability to manage conflicting work and family responsibilities has been overlooked. Yet, access to flexible hours, homeworking, and short-term leave options may help caregivers stay employed (Anand et al., 2022; Hill et al., 2020; Niimi, 2021). Most empirical studies that have looked at how working conditions may moderate the impact of care on employment outcomes are affected by endogeneity. Indeed, the decision to engage in care is linked to employment status, with part-time or nonemployed individuals being more likely to engage in care roles (Carmichael & Charles, 2003). Also, these studies do not usually consider all confounding factors affecting the propensity to care and engage in paid work (Heitmueller, 2007).

This study aims to fill these gaps by examining employment responses to a partner’s disability onset and how this relationship is moderated by working conditions, using a novel approach. We build on the econometric literature that addresses the endogeneity of work and care decisions by assessing how individuals’ work transitions are affected by a sudden, unexpected change in care responsibilities due to a partner’s ill health (Jolly & Theodoropoulos, 2021; Lee, 2020; Macchioni Giaquinto et al., 2022). We refer to this as a “shock” and develop a new measure based on the onset of difficulties with activities of daily living (ADL) and/or instrumental activities of daily living (IADL). This approach allows us to study the causal impact of care on employment outcomes without relying on self-reported measures of care, thus avoiding “anticipation bias” (Jeon & Pohl, 2017). By combining it with preprocessing methods (coarsened exact matching and entropy balancing), we increase confidence in our estimates showing causal effects. We explore how gender and working conditions, specifically psychosocial job demands and job satisfaction, moderate the effect of “care shocks” on paid work. We consider a broader set of responses to a “care shock”—not just leaving paid employment, but also working part-time, changing jobs, and looking for a new job.

## Background

### Spousal Caregiving and Employment Behaviors

In the United Kingdom, 34% of carers were spouses in 2004–2006, similar to the 16-country OECD average (Colombo et al., 2011). An extensive body of research suggests that spousal caregivers—as co-resident caregivers involved in high-intensity caregiving—are particularly likely to reduce their labor force participation and working hours (Carmichael et al., 2010; Carr et al., 2018; Heitmueller, 2007). Economists theorize that spousal caregiving may affect employment through substitution or income effects. That is, caregiving reduces the amount of time available, leading to reduced work hours or leaving paid work (i.e., substitution effect). Conversely, carers may increase labor supply to cover caregiving costs (i.e., income effect). The latter is especially salient for spousal carers, who may face financial pressures such as securing retirement, maintaining benefits, and funding education (Lima et al., 2008). The balance between these effects determines caregiving’s impact on labor market participation: employment increases if the income effect outweighs the substitution effect (Heitmueller, 2007).

Beyond the mechanisms hypothesized by economists, other factors play an important role in the impact of caregiving on employment decisions. Paid employment not only provides an income but also other benefits, such as social capital and symbolic resources, as being employed is socially valued (Damaske, 2011). Moreover, employment may serve as respite, providing time off caregiving responsibilities (Utz et al., 2012). Nevertheless, carers face wage discrimination in the workplace because of their caregiving commitments and the need for greater work flexibility (Heitmueller & Inglis, 2007). Additionally, caregiving demands may offer an opportunity to justify leaving an unsatisfactory job characterized by adverse working conditions (Damaske, 2011; Pavalko & Artis, 1997).

Finally, because caregiving involves emotional and physical demands, it can affect employment through its impact on carers’ health. Stress models posit that this health decline results from primary stressors associated with caregiving tasks and secondary stressors resulting from the intersection of these tasks with work, family, and other areas of the caregiver’s life (Pearlin & Zarit, 1993).

### Partners’ Health Deterioration: An Event to Exploit for Better Assessing the Effect of Spousal Caregiving on Employment Behaviors

To date, the literature on the impact of caregiving on labor market participation has several limitations. First, it mostly relies on self-reported caregiving measures. Research shows that self-identifying as a carer does not consistently align with actual caregiving activities (Beatie et al., 2021; Carduff et al., 2014). Moreover, self-reports of unpaid caregiving activities are subject to variations based on survey design and question-wording (Corden & Hirst 2011). Tasks considered as caregiving also differ based on normative expectations tied to gender roles, with males viewing housework as caregiving, whereas women focus on new tasks related to their partner’s impairment (Morgan et al., 2021). Caregiving may be better captured by the partner’s level of functional impairment, distinguishing IADLs and ADLs that imply different types of assistance to meet needs (Bertogg & Strauss 2020). Most of the literature is also limited by endogeneity bias and unobserved heterogeneity—as noted earlier.

Recent studies have overcome some of these limitations by examining individuals’ employment transitions following their partner’s health deterioration, commonly referred to as a “health shock.” These “health shocks” include events such as a cancer diagnosis (Jeon & Pohl, 2017), stroke and heart attacks (Fadlon & Nielsen, 2021; Jeon et al., 2019; Macchioni Giaquinto et al., 2022) or the onset of a work-limiting disability (Jolly & Theodoropoulos, 2021; Lee 2020). These studies have yielded mixed results, with some finding significant declines in employment following such events (Jeon and Pohl 2017; Jolly & Theodoropoulos, 2021; Lee 2020), whereas others report no significant change in labor force participation (Fadlon & Nielsen, 2021; Jeon et al., 2019; Macchioni Giaquinto et al., 2022).

However, it is questionable whether these studies truly capture the impact of caregiving on employment, as not all of these forms of “health shocks” necessarily lead to increased caregiving demands. For example, although some cancer diagnoses may result in significant care needs, others may not involve functional impairments requiring assistance with activities of daily living. To address this, we focus on transitions where an individual’s ability to perform everyday tasks—such as dressing, bathing, moving around, or managing household chores—becomes significantly impaired, indicating a higher likelihood of needing care (Ueshima et al., 2020). We define this transition as a “care shock” for the partner, which enables us to examine the labor market implications of a potential increase in care demands.

### The Importance of Working Conditions in the Effect of Caregiving on Employment Behaviors

Job characteristics may play a significant role in moderating the impact of care on employment. Stress models suggest that carers quitting work may result from a lack of coping resources, such as support networks and workplace policies (Pearlin & Zarit 1993). In the United Kingdom, carers report that flexible working practices (flexible hours, homeworking, part-time work, and short-term leave options) would help them stay in paid work (Carers UK, 2019). A few studies have investigated this, yielding mixed results. In the United States, women providing care to ill or disabled family members are more likely to stay employed if they have access to jobs with flexible hours, and to paid or unpaid family leave (Anand et al., 2022; Pavalko & Henderson 2006). Similar findings have been observed in Austria (Schneider et al., 2012) and Japan (Niimi, 2021). In Australia, factors such as holding a nonpermanent job, working in a smaller firm, and perceiving poor job security, increase the likelihood of leaving paid work after taking on care responsibilities (Hill et al., 2020). Other studies show no influence of job flexibility on the employment–caregiving interaction (Henz 2006).

Except for Anand et al. (2022), who uses the “health shock” approach, most of these studies are prone to endogeneity bias. Moreover, they largely focus on carer’s leave and workplace policies, ignoring broader working conditions. Yet, different dimensions of working conditions may influence work/caregiving reconciliation strategies. High job demands—such as long hours, tight deadlines and heavy workloads—pose significant challenges for carers in reconciling work and family roles and may lead them to change their employment circumstances (Bowling et al., 2015). However, workplace resources—such as flexible working hours, autonomy, and supportive colleagues—can help mitigate these challenges (Demerouti et al., 2005) and enable carers to better juggle dual responsibilities (Spann et al., 2020). In the United Kingdom, carer benefits such as carer’s allowance and statutory leave, which are primarily government entitlements, act as resources to provide financial support and time off for carers. However, high job demands and limited workplace support may prevent carers from taking full advantage of these benefits, for example, due to time constraints or fear of career repercussions (Brimblecombe et al., 2020). The combination of high demands and limited resources often leads to job dissatisfaction, which may result in job change or withdrawal from the labor force as caring responsibilities increase (Sousa-Poza, 2007).

## Method

### Data

We employ the English Longitudinal Study of Ageing (ELSA), an ongoing multidisciplinary longitudinal biennial survey of individuals aged 50 and over in private households. In the first wave collected in 2002/03, around 12,000 respondents were recruited to provide a representative sample of the population aged 50 and over living in private households in England (household response rate was 70%). More details of the survey’s sampling frame, methodology, and questionnaires are reported elsewhere (https://www.elsa-project.ac.uk).

### Measures

#### Identification of “care shocks”

Individuals whose partners report the onset of difficulties in at least one ADL or IADL between two consecutive waves (*n* = 1020) are defined as experiencing a “care shock.” We compare changes in employment outcomes between respondents who experienced this “care shock” between *t* and *t* + 1 and those who did not. The ADL items include (a) dressing, (b) walking across a room, (c) bathing or showering, (d) difficulty eating, (e) getting in and out of bed, (f) using the toilet. The IADL items include (a) using a map, (b) recognizing a physical danger, (c) preparing a hot meal, (d) shopping for groceries, (e) making telephone calls, (f) communicating, (g) taking medications, (h) doing work around the house or garden and (i) managing money. We consider the onset of a partner’s disability as a “care shock” as ADLs and/or IADLs are good predictors of care provision (Ueshima et al., 2020).

We restrict the sample to married or cohabiting individuals observed in at least two consecutive waves (*t* and *t* + 1) across the nine waves. Furthermore, we limit our focus to participants aged 50–69, in paid work (self-employed or employed), and whose partners have no ADL/IADL limitation at time *t*. We define “treated individuals” as those whose partners experience the onset of an ADL or IADL disability between two consecutive waves. Respondents are categorized as “controls” if their partners do not report the onset of an ADL/IADL limitation during the observation period. Although treated individuals are only observed once, control individuals may contribute multiple observations across different pairs of waves (*t* and *t* + 1). Our final sample consists of 1,020 treated individuals and 12,810 controls (*N* = 13,830).

Our approach relies on conditional independence: without a partner’s disability onset, we are assuming that individuals’ employment outcomes would have been the same conditional on past outcomes and any confounding covariates (O’Neill et al., 2016). This requires data on factors influencing both the probability of a “care shock” and employment behaviors such as sociodemographic, health, and employment characteristics—information available in ELSA. In addition, by implementing matching techniques and adjusting the models for time-varying confounders and lagged employment outcomes (work hours, type of contract), we control for nontime-varying unmeasured characteristics that may be associated with employment outcomes at *t* + 1 (O’Neill et al., 2016).

#### Employment behaviors

Most past research focuses on a few work transitions (e.g., leaving employment or changing working hours), neglecting the wide variety of work experiences and transitions in later life (Fevang et al., 2012). We suggest that individuals may also adapt to a sudden change in caregiving by altering their working time, and/or by seeking a job more compatible with their caregiving responsibilities. Therefore, we build two variables to characterize various employment responses. The first variable indicates whether the partner or potential caregiver is: (a) employed full-time, (b) employed part-time, or (c) out of paid work. The second variable describes their status as follows: (a) in paid work with a new job, (b) in paid work and actively seeking a new job, (c) in paid work without a job change and not looking for a new job, or (d) out of paid work.

“Individuals out of paid work” comes from a question asking about their activities during the past month and refers to all those who did not report engaging in paid work or self-employment, unless they were temporarily away from work or waiting to begin a job. Additional questions were used to ensure they were not in other work-related arrangements (freelance, agency work). “Individuals working part-time” are those who reported working fewer than 35 hours per week. The self-reported working time question was only asked to those who did not report they were in paid work (as defined above). “Individuals who have a new job” are those who answered positively to the question “Is the job you had last time you were interviewed still your main job?” and those “looking for a new job” are identified from the question “Are you currently looking for a new job?.”

#### Working conditions

Information on working conditions is collected for individuals in paid work through a self-completion questionnaire. To measure psychosocial job demands and job satisfaction, we use the items “I am under constant time pressure due to a heavy workload” and “All things considered I am satisfied with my job.” Responses are measured on a four-point scale, and we combine “strongly agree” with “agree” and “strongly disagree” with “disagree” (the latter being the reference category). Supplementary Figures 1 and 2 in Supplementary Material show the distribution of the indicators for job demands and job satisfaction. Most of the missing information is for individuals who did not fill in the self-completion questionnaire, or because these questions were only included from Wave 2 onwards. Working individuals with missing working condition information do not differ in sociodemographic characteristics but are often self-employed.

### Empirical Strategy

#### Preprocessing step: coarsened exact matching and entropy balancing

To ensure that individuals in the treated and control groups share similar characteristics before their partner’s “care shock,” we follow Jones et al.’s (2020) and Macchioni Giaquinto et al.’s (2022) analytical strategy and apply coarsened matching and entropy balancing to a set of potential confounders, before running regression models. Coarsened exact matching (CEM) coarsens continuous covariates (grouping them into a limited number of categories) and then applies the exact matching algorithm. It creates a set of stratas, each with the same values on all covariates: units in a strata that contain at least one treated and one control unit are retained; units in the remaining strata are discarded from this sample. Weights, relative to the number of treated and control units in total and in the strata, are assigned to each unit. In contrast with other common matching methods, CEM ensures the balance of univariate and joint distribution of confounding covariates. However, because this requires *exact* matches, it is necessary to use a small number of potential confounders to avoid a sharp reduction in sample size (Iacus et al., 2012).

After CEM has improved the overlap between treatment and control groups by discarding extreme units, we run entropy balancing (EB) on the matched re-weighted data to further ensure balance on a larger set of confounders, including noncoarsened covariates (Hainmueller 2012). EB assigns weights to each sample unit to make the two groups as similar as possible for a set of potential confounders while remaining as close as possible to a set of uniform base weights. We use the weights obtained from the CEM as base weights in EB, and then the weights generated by the EB algorithm are retained for use in the final regression models.

We implement CEM and entropy balancing on the following variables at *t*: age, sex, highest educational level achieved, number of children, equivalized household income, work hours, employment contract (i.e., fixed term, permanent, self-employed), number of ADLs and IADLs, depressive symptoms (assessed with the Center for Epidemiologic Studies Depression Scale [CES-D]), and partner’s age and diagnosis of at least one of the following conditions: heart attack, stroke, diabetes, chronic lung disease, cancer, Parkinson’s disease, cataract, fractured hip, dementia, any psychological problem, and arthritis. These variables capture characteristics likely to influence the impact of a “care shock” on employment outcomes and differ between respondents who report an ADL/IADL onset and those who do not (Cullati et al., 2014; Leopold, 2018; Lima et al., 2008; Meyler et al., 2007; Turek et al., 2024).

#### Modeling

After running CEM and EB, we run a series of models on the matched data. First, we run logistic regression to estimate the average treatment effect on the treated (ATT) of the partner’s IADL and/or ADL onset, along with alternative measures of “care shocks,” on self-reported caregiving provision. This way, we verify that it captures an increase in care and can be characterized as a “care shock.” Second, we run two multinomial logistic regressions to estimate the ATT of the “care shock” on employment behaviors. The first model estimates the probability of being in full-time work vs part-time work vs out-of-paid work. The second estimates the probability of being in paid work without a job change vs in paid work with a new job vs actively seeking a new job vs out-of-paid work. Then, to estimate the variation of the ATT on the treated with gender and working conditions (i.e., job demands and job satisfaction), we add interaction terms between treatment and gender, and between treatment and the two working condition items, in these multinomial models. To interpret these results as moderation effects, we ensure the balance between the treated and control groups across each level of the moderating variables, using coarsened exact matching and entropy balancing.

All models include the treatment variable indicating whether individuals’ partners experienced an ADL and/or IADL onset, and are adjusted for covariates used in the CEM and EB processes—listed above—to ensure a doubly robust approach (Ho et al., 2007). Preprocessing methods make results less sensitive to model misspecification, as balancing the distribution of covariates between the control and treated groups brings the treatment closer to being independent of confounding covariates.

#### Sensitivity analyses

We test the robustness of our results to alternative specifications. First, we repeat our analyses using alternative measures of “care shocks,” including the onset of one ADL (*n* = 612), one IADL (*n* = 643), or 2 IADLs and/or ADLs (*n* = 336). Second, we replicate our analyses using one of the “health shock” measures previously used in the literature: the report of a doctor-diagnosed condition including cancer, stroke, or heart attack (*n* = 518). Third, we test the robustness of our results by restricting the analysis to individuals below their state pension age. Finally, we also address changes in health as a time-varying confounder by excluding those who developed an IADL and/or ADL and adjusting for CES-D transitions between *t* and *t* + 1. Detailed results for these analyses are provided in Supplementary Material.

## Results

### Description of Individuals Who Experience a “Care Shock”

Treated individuals are those whose partners first report at least one ADL and/or IADL limitation onset between two consecutive waves. Supplementary Table 1 shows that between 6% and 9% of the sample report the onset of an ADL and/or IADL between each pair of consecutive waves. Among respondents with the onset of a functional limitation, 36% report an ADL, 40% an IADL, and 23% both an ADL and IADL. Supplementary Table 2 shows the distribution of partner ADLs and IADLs for the treated group (i.e., respondents who experienced a “care shock”). The most reported items are difficulties in dressing (41%) and in taking medications (39%).

Table 1 shows the descriptive characteristics of respondents who experience a “care shock” and those who did not (along with partner characteristics). People who experience a “care shock” are in lower socio-economic status groups: they are less likely to be in the managerial or professional class (33% vs 40%), to report tertiary education (20% vs 26%), and have a lower average income. They are also less likely to be self-employed (16% vs 19%), and report a higher mean number of IADLs/ADLs or diagnosed diseases in the wave before treatment. Their partners who experience an ADL/IADL onset, are older, report lower levels of education, and are more likely to have reported a doctor-diagnosed disease in the previous wave.

**Table 1.** Characteristics of Individuals Who Experienced A “Care Shock” In Comparison With the Rest of the Sample

Characteristic	No care shock *n* = 12,810		Care shock *n* = 1020		*p* Value
	Mean (SE)	%	Mean (SE)	%	
Age	59.2 (4.5)		59.7 (4.5)		<.001
Household equivalent income (*t*)	22,240.1 (19,082.1)		19,701.0 (15,763.7)		<.001
Number of ADL and/or IADL (*t*)	0.12 (0.59)		0.18 (0.71)		<.001
CES-D score (*t*)	0.93 (1.50)		1.11 (1.60)		<.001
Partner age	59.3 (5.9)		60.7 (6.5)		<.001
Partner number of diagnosis (*t*)	1.45 (0.73)		1.89 (1.00)		<.001
Sex					.11
Female		48.5%		46.0%	
Male		51.5%		54.0%	
Education					<.001
Less than upper sec.		14.4%		21.5%	
Upper sec/vocational training		59.7%		58.3%	
Tertiary		25.9%		20.2%	
Social origin					<.001
Other, armed forced or never worked		19.9%		22.1%	
Managers and professionals, clerks		40.3%		32.8%	
Semi-skilled and unskilled manual		9.2%		9.4%	
Skilled manual and service workers		30.7%		35.8%	
Working time (*t*)					.7
Full-time		40.9%		41.5%	
Part-time		59.1%		58.5%	
Employment status (*t*)					.013
Fixed term job		5.5%		5.5%	
Permanent job		75.1%		78.8%	
Self-employed		19.4%		15.7%	
Household composition (*t*)					<.001
No child in the household		62.5%		68.1%	
One child or more		37.5%		31.9%	
Partner’s education					<.001
Less than O-level		28.5%		40.6%	
O-level		32.4%		29.1%	
Higher than A level		39.1%		30.3%	

*Note*. ADL = activities of daily living; CES-D = Center for Epidemiologic Studies Depression Scale; IADL = instrumental activities of daily living.

As noted above, we use CEM and EB to improve the balance between treatment and control groups in terms of covariates. The results before CEM and EB, and post-CEM and EB are in Supplementary Table 3, showing that balancing is achieved for all covariates after CEM and EB. Although these techniques strengthen the validity of our results by minimizing confounding within the matched sample, they also limit the generalizability of our findings to the wider population. This is because individuals who do not have a matched counterpart on the basis of the selected covariates are excluded, resulting, for example, in the under-representation of individuals from higher socio-economic backgrounds or with younger partners.

### The Effect of a Partner’s ADL or IADL Onset on Self-Reported Caregiving Provision

Before looking at the impact of care shocks on employment, we first check how far our measure of “care shocks” predicts self-reported caregiving provision. This is shown in Table 2, which describes the ATT (calculated as the average marginal effect on the balanced sample) and the relative size effect—not just of our main measure, but of four alternative sensitivity analyses. “Relative size effect” compares the size of effects relative to their prevalence, computed as 100 * ATT/Conterfactual outcome for the control group (Macchioni Giaquinto et al., 2022).

**Table 2.** The Average Treatment Effects on the Treated (ATTs) of Various “Care Shock” Measures on the Probability of Providing Care At *t*

“Care shock” measure	Estimate	Confidence interval	*p* Value	Relative size effect
1 ADL/IADL onset	0.07	[0.05, 0.10]	<.001	60.1
*Conditions approach*				
Heart attack/stroke/cancer	0.05	[0.01, 0.08]	.01	33.5
*Alternative specifications*				
1 ADL onset	0.09	[0.05, 0.12]	<.001	66.6
1 IADL onset	0.10	[0.07, 0.14]	<.001	89.5
2 ADL/IADL onset	0.17	[0.12, 0.22]	<.001	149.54

*Note*. ADL = activities of daily living; IADL = instrumental activities of daily living.

Individuals whose partners report the onset of an IADL or an ADL limitation have a 0.07 point (i.e., 7 percentage points) increase in the probability of reporting caregiving in the past week, which translates to a 60% relative increase in the likelihood of providing caregiving. This increase highlights the significant impact of partner limitations on caregiving duties and justifies its use as an indicator of care shocks. The onset of a stroke, cancer, or heart attack yields a comparable effect on self-reported caregiving. However, as anticipated, the onset of at least two IADLs or ADLs emerges as a stronger predictor of self-reported care provision. Furthermore, we observe that the ATT of IADL onset on self-reported care surpasses that of ADL onset.

### The Effect of a Partner’s IADL or ADL Onset on Labor Market Outcomes

Table 3 shows the results of the models for the two sets of outcomes considered. Individuals who experience a care shock significantly reduce their labor force participation by transitioning out of paid work. On average, individuals’ likelihood of not being in paid work the year after the care shock increases by 0.025 (i.e., 2.5 percentage points; 95% CI [0.002, 0.049]), an 8% increase relative to the mean counterfactual outcome. At the same time, their probability of working full-time postshock decreases by 0.03 (95% CI [−0.054, −0.006]). We do not observe a significant increase in individuals’ part-time employment, job change, or job change intention.

**Table 3.** The Average Treatment Effect on the Treated (ATT) on Labor Market Outcomes At Time *t*

Outcome	Estimate	Confidence interval	*p* Value	Relative size effect
***Model 1—Workforce participation (n = 11,248)***				
Full-time employed	−0.03	[−0.054, −0.006]	.016	−8.943
Part-time employed	0.004	[−0.021, 0.03]	.730	1.326
Not in paid work	0.025	[0.002, 0.049]	.035	7.617
***Model 2—Job changes (n = 11,588)***				
Looking for a new job	0.002	[−0.009, 0.013]	.746	0.744
New job	0	[−0.023, 0.024]	.968	0.19
In paid work, no new job nor looking for a new job	−0.027	[−0.057, 0.004]	.086	−10.733
Not in paid work	0.024	[0.001, 0.048]	.037	9.799

*Notes*: The ATT is calculated by averaging the differences between the actual and predicted counterfactual outcomes over the distribution of treated individuals in the matched sample. The ATT is adjusted for the observable confounders listed on page 12.

*Source*: ELSA, Waves 1–9.

This response to the “care shock” appears to be homogenous for men and women, as the models with interaction terms show no significant difference in the effect of a “care shock” on employment outcomes by gender (Supplementary Table 4). In sensitivity analyses, the results are similar when we restrict the analysis to individuals below their state pension age (Supplementary Table 5). The ATTs of alternative care shock measures (ADL only, 2 IADL/ADLs, and health shocks) on employment outcomes display similar patterns, except for health shocks, which also have more imprecise estimates and larger confidence intervals (Supplementary Tables 6–9).

### Does It Vary With Job Demands and Job Satisfaction?

Tables 4 and 5 show how the employment responses to “care shocks” vary by working conditions. The first table shows significant variation in ATTs by job demands, specifically regarding whether individuals reported experiencing pressure due to heavy workloads the wave before the “care shock.” Indeed, individuals who report not having to work under pressure in their jobs demonstrate a 0.032 (nonsignificant) decrease in their probability of being out of paid work after the shock (95% CI [−0.071, 0.007]). Conversely, those who report working under pressure show a significant 0.061 increase in their likelihood of exiting paid employment after the shock (95% CI [0.014, 0.109]), equivalent to an 18% increase compared with those who did not experience the shock.

**Table 4.** The Average Treatment Effects on the Treated (ATTs) of a ‘Care Shock’ on Employment Outcomes Depending on Whether Individuals Had Pressure in Their Job At *t*

Outcome	Work pressure (t-1)	Estimate	Confidence Interval	*p* Value	Relative size effect	Second-diff estimate	Confidence interval
***Model 1—Workforce participation (n = 7,311)***							
Full-time	No work pressure	−0.001	[−0.036, 0.034]	.944	−0.376	0.031	[−0.028, 0.089]
	Work pressure	−0.032	[−0.079, 0.015]	.184	−9.576		
Part−time	No work pressure	0.033	[−0.009, 0.075]	.126	9.92	0.063	[0.001, 0.124]
	Work pressure	−0.03	[−0.075, 0.015]	.198	−8.872		
Not in paid work	No work pressure	−0.032	[−0.071, 0.007]	.108	−9.544	−0.093	[−0.155, 0.032]
	Work pressure	0.061	[0.014, 0.109]	.011	18.448		
***Model 2—Job changes (n = 7,311)***							
Looking for new job	No work pressure	−0.001	[−0.015, 0.012]	.841	−0.426	−0.012	[−0.042, 0.018]
	Work pressure	0.011	[−0.016, 0.037]	.425	3.225		
New job	No work pressure	0.022	[−0.015, 0.06]	.244	6.699	0.059	[0.005, 0.114]
	Work pressure	−0.037	[−0.077, 0.002]	.066	−11.117		
In paid work, no new job nor looking for a new job	No work pressure	0.012	[−0.037, 0.061]	.635	3.552	0.043	[−0.033, 0.119]
	Work pressure	−0.031	[−0.089, 0.027]	.294	−9.349		
Not in paid work	No work pressure	−0.033	[−0.07, 0.005]	.087	−9.825	−0.09	[−0.15, −0.03]
	Work pressure	0.057	[0.011, 0.104]	.016	17.241		

*Note*s: The ATT is calculated by averaging the differences between the actual and predicted counterfactual outcomes over the distribution of treated individuals in the matched sample. The ATT is adjusted for the observable confounders listed on page 12.

*Source*: ELSA, Waves 1−9.

**Table 5.** The Average Treatment Effects on the Treated (ATTs) of a ‘Care Shock’ on Employment Outcomes Depending on Whether Individuals Were Satisfied With Their Job At *t*

Outcome	Job satisfaction (*t*−1)	Estimate	Confidence Interval	*p* Value	Relative size effect	Second-diff estimate	Confidence interval
***Model 1—Workforce participation (n = 7,703)***							
Full time	Satisfied with their job	−0.012	[−0.041, 0.018]	0.445	−3.452	0.065	[0.005, 0.125]
	Not satisfied with their job	−0.077	[−0.139, −0.014]	0.016	−22.981		
Part time	Satisfied with their job	0.017	[−0.016, 0.05]	0.319	5.077	0.005	[−0.052, 0.061]
	Not satisfied with their job	0.012	[−0.043, 0.067]	0.664	3.64		
Not in paid work	Satisfied with their job	−0.005	[−0.037, 0.026]	0.735	−1.626	−0.07	[−0.135, −0.005]
	Not satisfied with their job	0.064	[0, 0.129]	0.05	19.341		
***Model 2—Job changes (n = 7,703)***							
Looking for new job	Satisfied with their job	0	[−0.014, 0.013]	0.976	−0.062	−0.025	[−0.071, 0.021]
	Not satisfied with their job	0.025	[−0.024, 0.073]	0.324	7.352		
New job	Satisfied with their job	0.023	[−0.005, 0.052]	0.108	7.017	0.023	[−0.023, 0.069]
	Not satisfied with their job	0	[−0.046, 0.046]	0.991	0.079		
In paid work, no new job	Satisfied with their job	−0.015	[−0.054, 0.024]	0.44	−4.589	0.067	[−0.004, 0.138]
	Not satisfied with their job	−0.083	[−0.155, −0.01]	0.025	−24.776		
Not in paid work	Satisfied with their job	−0.008	[−0.038, 0.023]	0.611	−2.366	−0.066	[−0.129, −0.002]
	Not satisfied with their job	0.058	[−0.006, 0.122]	0.077	17.345		

*Note*s. The ATT is calculated by averaging the differences between the actual and predicted counterfactual outcomes over the distribution of treated individuals in the matched sample. The ATT is adjusted for the observable confounders listed on page 12.

*Source*: ELSA, Waves 1–9.

The second table shows significant variation in ATTs by job satisfaction. People who experience a “care shock” show unequal chances of being out of paid work depending on whether they are satisfied with their job before the “care shock.” Individuals who report not being satisfied with their jobs exhibit a 0.064 increase in their likelihood of being out of paid work postshock (95% CI [0, 0.0129]), whereas those who report being satisfied with their job show no significant variation in the probability of not being in paid work (95% CI [−0.037, 0.026]).

Thus, our findings suggest that job demands and job satisfaction significantly moderate employment responses to a “care shock.” In sensitivity analyses with alternative “care shock” measures (Supplementary Tables 10–19), we observe similar significance patterns, except for the onset of a stroke/cancer/heart attack, which also has more imprecise estimates and larger confidence intervals.

## Discussion

To the best of our knowledge, this is the first study to assess how the impact of care on labor market behavior is moderated by working conditions, particularly job demands and job satisfaction. Previous research mostly focuses on job flexibility and access to paid leave but does not explore other dimensions of working conditions. We investigate the effect on paid work of a sudden onset of an ADL or IADL in one’s partner—which we define as a “care shock”—to tackle endogeneity and self-report bias in care measures. In contrast to previous studies, we examine a wider range of labor market responses to the onset of caregiving, related to workforce participation status, working hours, job changes, and job change intentions.

Our study shows several novel results. First, we find that “care shocks” significantly increase the likelihood of individuals being out of paid work whereas they decrease the probability of working full-time. Their impact on job changes or job change intentions is not significant or large at conventional significance levels. This indicates that individuals respond to the onset of care responsibilities largely by exiting paid work from full-time jobs. This is consistent with some previous research documenting the negative impact of partners’ “health shocks” – such as cancer diagnoses (Jeon & Pohl, 2017), the onset of various chronic conditions (McGeary 2009), and work-limiting disabilities (Lee, 2020)—on both the likelihood and intensity of labor force participation. However, our findings contrast with those of other studies that report no significant impact of such health transitions on employment (Fadlon & Nielsen, 2021; Jeon et al., 2019; Macchioni Giaquinto et al., 2022). They also contradict research suggesting that spouses increase their labor supply to compensate for the income loss resulting from their partner’s ill health (García-Gómez et al., 2013). This may be because in comparison to health diagnoses, focusing on partners’ functional health decline in the form of limitations in activities such as dressing, preparing meals, or managing medication (ADLs and IADLs) allows us to better capture care demands and their impact on employment. Furthermore, the age range of our study population may contribute to this difference, as financial security among older individuals may be less reliant on health status compared with younger couples (Acuña et al., 2019). Our findings underscore the importance of studying late careers at the couple level, as individuals’ opportunities and constraints are linked to those of their partners, particularly concerning health-related factors (Carr 2018).

Surprisingly, our results do not reveal significant gender differences in employment responses to a care shock, which contrasts with previous UK findings showing that care disproportionately affects women’s employment and working hours (Carr et al., 2018). This difference may be because our IADL/ADL-based measure eliminates the reporting bias of self-reported care indicators, where women identify as carers at a higher threshold of caregiving effort than men. This is consistent with recent findings showing that men and women respond to the onset of a partner’s serious illness by performing similar household and care tasks (Langner & Furstenberg, 2020).

We also find that individuals’ labor market responses to a “care shock” are linked to job demands and job satisfaction. Indeed, people who report “having to work under pressure because of a heavy workload” prior to a “care shock,” as well as those who report low job satisfaction, exhibit a higher likelihood of leaving paid work. For those who did not report work pressure or who reported being satisfied with their job, the “care shock” does not affect the probability of leaving paid work. This underscores the potential benefits of adapting working conditions, by reducing job demands, to facilitate individuals’ continued attachment to the labor market in later stages of their careers. Although past research suggests the importance of caregiving characteristics, gender, and socioeconomic status in moderating the effect of care on health (Riekhoff & Vaalavuo 2021), our results show the importance of considering the heterogeneity of jobs. Our study adds to the body of research on the importance of workplace policies, such as access to paid leave and job flexibility, in supporting individuals facing care demands (Anand et al., 2022).

However, this study has some limitations. First, the “care shock” may been anticipated. We imperfectly control for diseases developed by partners in the years preceding the onset of ADL and IADLs, and people may then have adjusted their labor market status accordingly. Consequently, we likely miss individuals who have already exited the workforce to provide care to their partners, thereby introducing attenuation bias into our analysis. We may also underestimate the impact of “care shocks” on employment outcomes as individuals whose partners experience the most severe “care shocks” may have a higher attrition rate.

Also, due to sample size constraints, we did not capture responses to the “care shock” at *t* + 1 or *t* + 2. Individuals may adapt their employment behavior later following the “care shock,” or individuals may reenter the labor market, which we do not observe. We are then not able to capture certain adaptations to increased care demands, which may become apparent in a longer-term trajectory. Our analyses may also miss some other forms of employment adaptations. For example, we only observe whether individuals change jobs without characterizing the direction of these transitions. Examining whether individuals move to a more flexible job or a position with fewer responsibilities could provide us with a better understanding of individuals’ responses to care constraints. We are also unable to capture greater heterogeneity in individuals’ responses to care shocks. For example, due to data constraints, we were not able to explore variations in the impact of a “care shock” on paid work by detailed age groups. This is likely to be important, as individuals’ responses to increased care responsibilities may differ based on their proximity to retirement.

Moreover, the consequences of a partner’s disability onset may extend beyond the quantity of care demand. For example, individuals whose partners become ill may experience a decline in their mental health, which may affect their likelihood of leaving the labor force, working part-time, or changing jobs. These individuals may also undergo changes that affect their employment decisions, such as reevaluating life priorities or wanting to spend more time with a partner as their health declines. Therefore, the effect we observe may not only reflect the impact of caregiving on employment, but may also be influenced by mental health and other time-varying, unobserved confounders. To address this, we conducted sensitivity analyses by excluding individuals who experienced a health deterioration between *t* and *t* + 1. The effect of a “care shock” on the probability of exiting paid work remained significant for individuals with high job demands or job dissatisfaction, suggesting that care constraints are primarily driving these individuals’ work exits (Supplementary Tables 20–22). Further research using modeling strategies that better account for unobserved heterogeneity is needed to clarify the moderating role of working conditions in the impact of care on employment.

Furthermore, our analyses focus on the onset of partner disability (i.e., the onset of any new difficulty between *t* and *t* + 1) as our approach relies on unanticipated health changes. However, it would be worth examining how individuals’ employment outcomes are affected by specific characteristics of their partners’ disability, such as the stage of the disability progression, whether the disability limits work or the type of functional limitation. For example, the impact of the onset of IADL/ADL limitations may differ from that of other transitions—such as an increase in the number of limitations. A partner may initially have difficulties with self-care tasks such as dressing or taking medication, but as the condition progresses they may face additional challenges with more complex tasks such as navigating transport. These stages of functional decline can have distinct effects on employment outcomes. Individuals may also be affected differently by the onset of their partner’s disability, depending on whether the disability limits their partner’s ability to work. Sensitivity analyses indicate that individuals whose partners experience an ADL/IADL onset and are no longer employed are more likely to leave paid work themselves than those whose partners remain employed despite the onset (Supplementary Table 23). Further analysis using larger data sets and alternative measures of “care shock,” including data on work-limiting disabilities, would improve our understanding of how care demands affect labor market outcomes.

Last, we rely on self-reported working conditions, which may introduce bias. Individuals’ reports of their working conditions are influenced by various factors, including their health status(Eguchi et al., 2012). This disparity in health status among those with adverse working conditions may explain why they are more inclined to leave their jobs. Replicating this study using job-exposure matrices could help reduce reporting bias and endogeneity (Solovieva et al., 2014). Also, our data only allow for the measurement of job satisfaction and job demands. Yet, other dimensions of working conditions may play an important role and should be explored. For example, job control—the degree of autonomy or decision-making authority that individuals have over their work tasks and environment (Karasek 1979)—could impact people’s ability to manage constraints linked to care demand. Further studies should investigate these dimensions to provide a broader understanding of how working conditions influence employment decisions in the context of care responsibilities.

## Supplementary Material

gbae208_suppl_Supplementary_Materials

## Data Availability

Researchers can download ELSA data from all waves, from the UK Data Service. For more information, please visit https://www.elsa-project.ac.uk/accessing-elsa-data.
