# Supplementary material for: Employment Responses to a Partner’s Disability Onset (“Care Shocks”): Do Working Conditions Matter?
Source: J Gerontol B Psychol Sci Soc Sci. 2024 Dec 27;80(4):gbae208. doi: 10.1093/geronb/gbae208 (PMC11898210; doi:10.1093/geronb/gbae208)
Supplement: gbae208_suppl_Supplementary_Materials [file gbae208_suppl_supplementary_materials.docx]

***The Journals of Gerontology, Series B: Psychological Sciences and Social Sciences* Supplementary Material: Beaufils, Geiger, & Glaser. Employment responses to a partner’s disability onset (care shocks’): Do working conditions matter?**

Supplementary Figure 1: Distribution of job pressure in the entire sample


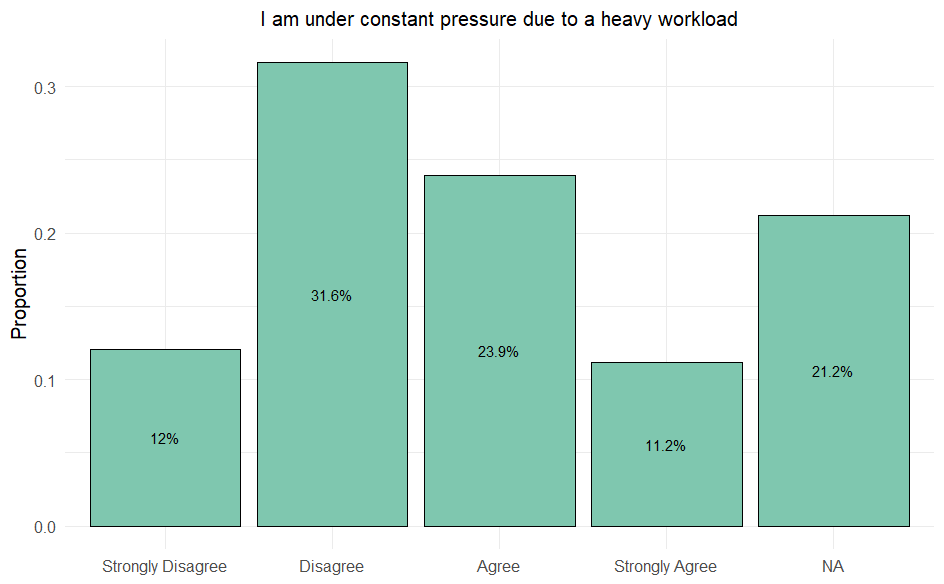


Supplementary Figure 2: Distribution of job satisfaction in the entire sample


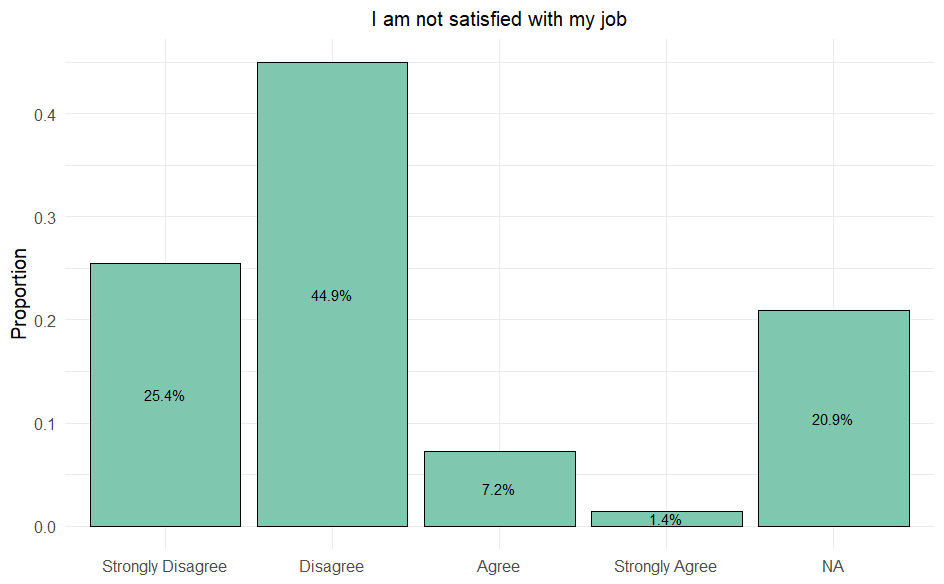


Supplementary Table 1: Number and frequency of individuals whose partner experienced an IADL/ADL onset across the different waves

| Wave | N | Frequency |
| --- | --- | --- |
| 2 | 190 | 9.19 |
| 3 | 116 | 7.31 |
| 4 | 140 | 7.34 |
| 5 | 151 | 6.87 |
| 6 | 141 | 7.85 |
| 7 | 111 | 6.22 |
| 8 | 100 | 6.85 |
| 9 | 71 | 6.90 |

Supplementary Table 2: Distribution of the first ADL and IADL reported among individuals who experienced a ‘care shock’

| ADL/IADL items | N | Frequency |
| --- | --- | --- |
| Dressing, including putting on shoes and socks | 418 | 40.98 |
| Taking medications | 399 | 39.12 |
| Bathing or showering | 176 | 17.25 |
| Using a map to figure out how to get around in a strange place | 157 | 15.39 |
| Getting in and out of bed | 139 | 13.63 |
| Shopping for groceries | 135 | 13.24 |
| Using the toilet, including getting up or down | 64 | 6.27 |
| Doing work around the house or garden | 58 | 5.69 |
| Communication (speech, hearing or eyesight) | 51 | 5 |
| Making telephone calls | 50 | 4.9 |
| Preparing a hot meal | 43 | 4.22 |
| Walking across a room | 31 | 3.04 |
| Difficulty eating, such as cutting up food | 29 | 2.84 |
| Recognising when you are in physical danger | 18 | 1.76 |
| Total | 1768 | 173.33 |

Supplementary Table 3: Covariate balance after entropy balancing and coarsened exact matching

|  | **Mean Difference (Sample)** | **Balance assessment (Sample)** | **Mean Difference (CEM)** | **Balance assessment (CEM)** | **Mean Difference (CEM and EB)** | **Balance assessment (CEM and EB)** |
| --- | --- | --- | --- | --- | --- | --- |
| Age | 0.12 | Not Balanced, >0.05 | -0.01 | Balanced, <0.05 | 0 | Balanced, <0.05 |
| Sex (Male) | 0.05 | Not Balanced, >0.05 | 0.00 | Balanced, <0.05 | 0 | Balanced, <0.05 |
| Education: Less than upper sec. | 0.19 | Not Balanced, >0.05 | 0.00 | Balanced, <0.05 | 0 | Balanced, <0.05 |
| Education: Upper sec/vocational training | -0.03 | Balanced, <0.05 | 0.00 | Balanced, <0.05 | 0 | Balanced, <0.05 |
| Education: Tertiary | -0.14 | Not Balanced, >0.05 | 0.00 | Balanced, <0.05 | 0 | Balanced, <0.05 |
| Household equivalent income (t) | -0.15 | Not Balanced, >0.05 | -0.16 | Not Balanced, >0.05 | 0 | Balanced, <0.05 |
| Number of children in the hh (t) | -0.12 | Not Balanced, >0.05 | 0.00 | Balanced, <0.05 | 0 | Balanced, <0.05 |
| Working time: Full-time (t) | -0.01 | Balanced, <0.05 | 0.00 | Balanced, <0.05 | 0 | Balanced, <0.05 |
| Self-employed (t) | -0.10 | Not Balanced, >0.05 | 0.00 | Balanced, <0.05 | 0 | Balanced, <0.05 |
| Number of ADL/IADL (t) | 0.09 | Not Balanced, >0.05 | 0.07 | Not Balanced, >0.05 | 0 | Balanced, <0.05 |
| Depressive episode (t) | 0.10 | Not Balanced, >0.05 | 0.00 | Balanced, <0.05 | 0 | Balanced, <0.05 |
| Partner’s number of ADL/IADL (t) | 0.50 | Not Balanced, >0.05 | 0.00 | Balanced, <0.05 | 0 | Balanced, <0.05 |
| Partner age | 0.24 | Not Balanced, >0.05 | 0.08 | Not Balanced, >0.05 | 0 | Balanced, <0.05 |

Supplementary Table 4: ATTs of a ‘care shock’ on employment outcomes depending on gender

| **Outcome** | **Gender** | **Estimate** | **Confidence Interval** | **p.value** | **RSE** | **Second-diff estimate** | **Confidence interval** |
| --- | --- | --- | --- | --- | --- | --- | --- |
| ***Model 1 - Workforce participation (n=11,295)*** | | |  |  |  |  |  |
| Full time | Female | -0.034 | [-0.065, -0.003] | 0.034 | -10.185 | -0.014 | [-0.06, 0.033] |
|  | Male | -0.02 | [-0.055, 0.014] | 0.248 | -6.121 |  |  |
| Not in paid work | Female | 0.019 | [-0.019, 0.058] | 0.32 | 5.842 | -0.009 | [-0.06, 0.041] |
|  | Male | 0.029 | [-0.004, 0.062] | 0.084 | 8.68 |  |  |
| Part time | Female | 0.014 | [-0.027, 0.056] | 0.495 | 4.343 | 0.023 | [-0.029, 0.075] |
|  | Male | -0.009 | [-0.04, 0.023] | 0.596 | -2.559 |  |  |
| ***Model 2 - Job changes (n= 11,558)*** | | |  |  |  |  |  |
| In paid work, no new job | Female | -0.029 | [-0.061, 0.002] | 0.065 | -11.76 | -0.002 | [-0.006, 0.001] |
|  | Male | -0.027 | [-0.058, 0.004] | 0.09 | -10.841 |  |  |
| Looking for new job | Female | 0.002 | [-0.009, 0.013] | 0.763 | 0.676 | 0 | [-0.002, 0.001] |
|  | Male | 0.002 | [-0.011, 0.015] | 0.751 | 0.811 |  |  |
| New job | Female | 0 | [-0.022, 0.021] | 0.974 | -0.142 | -0.001 | [-0.003, 0.002] |
|  | Male | 0 | [-0.024, 0.025] | 0.979 | 0.133 |  |  |
| Not in paid work | Female | 0.028 | [0.002, 0.054] | 0.036 | 11.226 | 0.003 | [0, 0.007] |
|  | Male | 0.025 | [0.001, 0.048] | 0.038 | 9.897 |  |  |

Supplementary Table 5: ATTs on labour market outcomes at time t (individuals aged below their state pension age - sensitivity analysis)

| **Outcome** | **Estimate** | **Confidence interval** | **p value** | **Relative size effect** |
| --- | --- | --- | --- | --- |
| ***Model 1 - Workforce participation (n=7,936)*** | | |  |  |
| Full time | -0.035 | [-0.065, -0.005] | 0.021 | -10.549 |
| Not in paid work | 0.026 | [0.002, 0.05] | 0.036 | 7.766 |
| Part time | 0.009 | [-0.018, 0.036] | 0.499 | 2.783 |
| ***Model 2 - Job changes (n=8,177)*** | | |  |  |
| In paid work, no new job | -0.032 | [-0.069, 0.004] | 0.079 | -12.958 |
| Looking for new job | 0.008 | [-0.008, 0.025] | 0.342 | 3.202 |
| New job | 0.002 | [-0.027, 0.031] | 0.905 | 0.707 |
| Not in paid work | 0.023 | [-0.001, 0.046] | 0.056 | 9.049 |

Supplementary Table 6: ATTs of 2 ADL and/or IADL on labour market outcomes at time t (sensitivity analysis)

| **Outcome** | **Estimate** | **Confidence interval** | **p value** | **Relative size effect** |
| --- | --- | --- | --- | --- |
| ***Model 1 - Workforce participation (n=8,727)*** | | |  |  |
| Full time | -0.049 | [-0.09, -0.008] | 0.02 | -14.743 |
| Not in paid work | 0.044 | [0.003, 0.086] | 0.037 | 13.297 |
| Part time | 0.005 | [-0.037, 0.046] | 0.819 | 1.445 |
| ***Model 2 - Job changes (n=8,974)*** | | |  |  |
| In paid work, no new job | -0.009 | [-0.06, 0.043] | 0.746 | -3.43 |
| Looking for new job | -0.013 | [-0.027, 0.001] | 0.059 | -5.29 |
| New job | -0.02 | [-0.058, 0.018] | 0.303 | -7.934 |
| Not in paid work | 0.042 | [0.001, 0.083] | 0.046 | 16.654 |

Supplementary Table 7: ATTs of 1 ADL on labour market outcomes at time t (sensitivity analysis)

| **Outcome** | **Estimate** | **Confidence interval** | **p value** | **Relative size effect** |
| --- | --- | --- | --- | --- |
| ***Model 1 - Workforce participation (n=10,373)*** | | |  |  |
| Full time | -0.036 | [-0.068, -0.005] | 0.023 | -10.887 |
| Not in paid work | 0.032 | [0.003, 0.062] | 0.032 | 9.693 |
| Part time | 0.004 | [-0.027, 0.035] | 0.804 | 1.194 |
| ***Model 2 - Job changes (n=10,684)*** | |  |  |  |
| In paid work, no new job | -0.014 | [-0.053, 0.024] | 0.462 | -5.789 |
| Looking for new job | -0.007 | [-0.019, 0.005] | 0.237 | -2.861 |
| New job | -0.007 | [-0.037, 0.022] | 0.619 | -2.996 |
| Not in paid work | 0.029 | [0, 0.058] | 0.048 | 11.646 |

Supplementary Table 8: ATTs of 1 IADL on labour market outcomes at time t (sensitivity analysis)

| **Outcome** | **Estimate** | **Confidence interval** | **p value** | **Relative size effect** |
| --- | --- | --- | --- | --- |
| ***Model 1 - Workforce participation (n=10,398)*** | | |  |  |
| Full time | -0.038 | [-0.068, -0.008] | 0.014 | -11.275 |
| Not in paid work | 0.032 | [0.002, 0.063] | 0.04 | 9.676 |
| Part time | 0.005 | [-0.026, 0.037] | 0.74 | 1.598 |
| ***Model 2 - Job changes (n=10,710)*** | |  |  |  |
| In paid work, no new job | -0.033 | [-0.071, 0.005] | 0.091 | -13.234 |
| Looking for new job | -0.001 | [-0.014, 0.013] | 0.936 | -0.221 |
| New job | 0.003 | [-0.025, 0.032] | 0.831 | 1.242 |
| Not in paid work | 0.031 | [0.001, 0.06] | 0.045 | 12.213 |

Supplementary Table 9: ATTs of a diagnosis (stroke, cancer, heart attack) on labour market outcomes at time t (sensitivity analysis)

| **Outcome** | **Estimate** | **Confidence interval** | **p value** | **Relative size effect** |
| --- | --- | --- | --- | --- |
| ***Model 1 - Workforce participation (n=9,386)*** | | |  |  |
| Full time | -0.005 | [-0.04, 0.031] | 0.802 | -1.371 |
| Not in paid work | 0.013 | [-0.02, 0.047] | 0.434 | 4.004 |
| Part time | -0.009 | [-0.044, 0.027] | 0.627 | -2.633 |
| ***Model 2 - Job changes (n=9,661)*** | | |  |  |
| In paid work, no new job | -0.02 | [-0.066, 0.027] | 0.407 | -7.875 |
| Looking for new job | -0.006 | [-0.02, 0.008] | 0.373 | -2.538 |
| New job | 0.013 | [-0.024, 0.05] | 0.491 | 5.236 |
| Not in paid work | 0.013 | [-0.021, 0.047] | 0.453 | 5.177 |

Supplementary Table 10: ATTs of a ‘care shock’ on employment outcomes depending on whether individuals had pressure in their job at t (individuals aged below their state pension age - sensitivity analysis)

| **Outcome** | **Work pressure (t-1)** | **Estimate** | **Confidence Interval** | **p.value** | **RSE** | **Second-diff estimate** | **Confidence interval** |
| --- | --- | --- | --- | --- | --- | --- | --- |
| ***Model 1 - Workforce participation (n=4,811)*** | | | | | | | |
| Full time | No work pressure | 0.005 | [-0.046, 0.056] | 0.846 | 1.514 | 0.059 | [-0.006, 0.125] |
|  | Work pressure | -0.054 | [-0.095, -0.014] | 0.009 | -16.299 |  |  |
| Not in paid work | No work pressure | -0.031 | [-0.073, 0.011] | 0.152 | -9.293 | -0.067 | [-0.123, -0.011] |
|  | Work pressure | 0.036 | [0, 0.073] | 0.052 | 10.885 |  |  |
| Part time | No work pressure | 0.026 | [-0.023, 0.075] | 0.3 | 7.779 | 0.008 | [-0.054, 0.069] |
|  | Work pressure | 0.018 | [-0.019, 0.055] | 0.342 | 5.414 |  |  |
| ***Model 2 - Job changes (N =4,811)*** | | | | | | | |
| In paid work, no new job | No work pressure | 0.026 | [-0.038, 0.089] | 0.427 | 7.682 | 0.078 | [-0.003, 0.159] |
|  | Work pressure | -0.052 | [-0.103, -0.002] | 0.044 | -15.698 |  |  |
| Looking for new job | No work pressure | 0.011 | [-0.014, 0.036] | 0.395 | 3.253 | -0.006 | [-0.041, 0.028] |
|  | Work pressure | 0.017 | [-0.006, 0.041] | 0.156 | 5.107 |  |  |
| New job | No work pressure | -0.003 | [-0.054, 0.049] | 0.917 | -0.821 | -0.004 | [-0.069, 0.062] |
|  | Work pressure | 0.001 | [-0.039, 0.041] | 0.969 | 0.238 |  |  |
| Not in paid work | No work pressure | -0.034 | [-0.074, 0.007] | 0.105 | -10.114 | -0.068 | [-0.123, -0.014] |
|  | Work pressure | 0.035 | [-0.001, 0.07] | 0.059 | 10.353 |  |  |

Supplementary Table 11: ATTs of a ‘care shock’ on employment outcomes depending on whether individuals were satisfied with their job at t (individuals aged below their state pension age - sensitivity analysis)

| **Outcome** | **Low satisfaction (t-1)** | **Estimate** | **Confidence Interval** | **p value** | **RSE** | **Second-diff estimate** | **Confidence interval** |
| --- | --- | --- | --- | --- | --- | --- | --- |
| ***Model 1 - Workforce participation (n=5,242)*** | | |  |  |  |  |  |
| Full time | Satisfied with their job | -0.007 | [-0.045, 0.031] | 0.72 | -2.083 | 0.021 | [-0.042, 0.083] |
|  | Not satisfied with their job | -0.028 | [-0.091, 0.036] | 0.39 | -8.33 |  |  |
| Not in paid work | Satisfied with their job | 0.013 | [-0.02, 0.045] | 0.44 | 3.843 | -0.008 | [-0.064, 0.047] |
|  | Not satisfied with their job | 0.021 | [-0.033, 0.076] | 0.446 | 6.365 |  |  |
| Part time | Satisfied with their job | -0.006 | [-0.042, 0.03] | 0.751 | -1.76 | -0.012 | [-0.066, 0.042] |
|  | Not satisfied with their job | 0.007 | [-0.046, 0.059] | 0.807 | 1.964 |  |  |
| ***Model 2 - Job changes (n=5,242)*** | | |  |  |  |  |  |
| In paid work, no new job | Satisfied with their job | -0.04 | [-0.088, 0.007] | 0.094 | -12.101 | 0.013 | [-0.067, 0.094] |
|  | Not satisfied with their job | -0.054 | [-0.134, 0.027] | 0.191 | -16.092 |  |  |
| Looking for new job | Satisfied with their job | 0.006 | [-0.014, 0.026] | 0.546 | 1.81 | -0.044 | [-0.107, 0.02] |
|  | Not satisfied with their job | 0.05 | [-0.016, 0.115] | 0.14 | 14.868 |  |  |
| New job | Satisfied with their job | 0.024 | [-0.014, 0.061] | 0.211 | 7.144 | 0.038 | [-0.009, 0.084] |
|  | Not satisfied with their job | -0.014 | [-0.058, 0.03] | 0.539 | -4.173 |  |  |
| Not in paid work | Satisfied with their job | 0.01 | [-0.021, 0.042] | 0.515 | 3.147 | -0.008 | [-0.062, 0.047] |
|  | Not satisfied with their job | 0.018 | [-0.035, 0.071] | 0.51 | 5.398 |  |  |

Supplementary Table 12: ATTs of 2 ADL and/or IADL on employment outcomes depending on whether individuals had pressure in their job at t (sensitivity analysis)

| **Outcome** | **Work pressure (t-1)** | **Estimate** | **Confidence Interval** | **p value** | **RSE** | **Second-diff estimate** | **Confidence interval** |
| --- | --- | --- | --- | --- | --- | --- | --- |
| ***Model 1 - Workforce participation (n=4,372)*** | | | | | | | |
| Full time | No work pressure | 0.005 | [-0.064, 0.074] | 0.885 | 1.532 | 0.078 | [-0.012, 0.169] |
|  | Work pressure | -0.073 | [-0.132, -0.014] | 0.015 | -21.973 |  |  |
| Not in paid work | No work pressure | -0.059 | [-0.133, 0.016] | 0.122 | -17.589 | -0.14 | [-0.237, -0.043] |
|  | Work pressure | 0.082 | [0.019, 0.144] | 0.011 | 24.466 |  |  |
| Part time | No work pressure | 0.054 | [-0.026, 0.133] | 0.187 | 16.057 | 0.062 | [-0.037, 0.161] |
|  | Work pressure | -0.008 | [-0.068, 0.051] | 0.784 | -2.493 |  |  |
| ***Model 2 - Job changes (n=4,372)*** | | | | | | | |
| In paid work, no new job | No work pressure | 0.04 | [-0.044, 0.124] | 0.354 | 11.994 | 0.091 | [-0.038, 0.221] |
|  | Work pressure | -0.051 | [-0.149, 0.046] | 0.301 | -15.449 |  |  |
| Looking for new job | No work pressure | -0.015 | [-0.033, 0.002] | 0.08 | -4.61 | -0.01 | [-0.054, 0.033] |
|  | Work pressure | -0.005 | [-0.044, 0.035] | 0.808 | -1.47 |  |  |
| New job | No work pressure | 0.023 | [-0.043, 0.088] | 0.501 | 6.755 | 0.076 | [-0.014, 0.165] |
|  | Work pressure | -0.053 | [-0.114, 0.007] | 0.086 | -15.932 |  |  |
| Not in paid work | No work pressure | -0.047 | [-0.112, 0.018] | 0.156 | -14.139 | -0.157 | [-0.263, -0.05] |
|  | Work pressure | 0.11 | [0.026, 0.193] | 0.011 | 32.851 |  |  |

Supplementary Table 13: ATTs of 2 ADL and/or IADL on employment outcomes depending on whether individuals were satisfied with their job at t (sensitivity analysis)

| **Outcome** | **Low satisfaction (t-1)** | **Estimate** | **Confidence Interval** | **p value** | **RSE** | **Second-diff estimate** | **Confidence interval** |
| --- | --- | --- | --- | --- | --- | --- | --- |
| ***Model 1 - Workforce participation (n=5,098)*** | | |  |  |  |  |  |
| Full time | Satisfied with their job | -0.035 | [-0.086, 0.015] | 0.17 | -10.582 | 0.093 | [-0.006, 0.191] |
|  | Not satisfied with their job | -0.128 | [-0.231, -0.024] | 0.016 | -38.343 |  |  |
| Not in paid work | Satisfied with their job | 0.012 | [-0.044, 0.067] | 0.683 | 3.468 | -0.131 | [-0.251, -0.01] |
|  | Not satisfied with their job | 0.142 | [0.021, 0.263] | 0.021 | 42.68 |  |  |
| Part time | Satisfied with their job | 0.024 | [-0.033, 0.081] | 0.416 | 7.115 | 0.038 | [-0.043, 0.119] |
|  | Not satisfied with their job | -0.014 | [-0.088, 0.059] | 0.698 | -4.337 |  |  |
| ***Model 2 - Job changes (n=5,098)*** | | |  |  |  |  |  |
| In paid work, no new job | Satisfied with their job | -0.015 | [-0.081, 0.051] | 0.657 | -4.498 | 0.127 | [-0.004, 0.259] |
|  | Not satisfied with their job | -0.142 | [-0.275, -0.009] | 0.037 | -42.654 |  |  |
| Looking for new job | Satisfied with their job | -0.015 | [-0.032, 0.003] | 0.097 | -4.444 | -0.035 | [-0.1, 0.03] |
|  | Not satisfied with their job | 0.02 | [-0.06, 0.101] | 0.619 | 6.125 |  |  |
| New job | Satisfied with their job | 0.021 | [-0.027, 0.069] | 0.39 | 6.338 | 0.022 | [-0.027, 0.07] |
|  | Not satisfied with their job | 0 | [-0.001, 0] | 0 | -0.128 |  |  |
| Not in paid work | Satisfied with their job | 0.009 | [-0.045, 0.062] | 0.75 | 2.604 | -0.114 | [-0.229, 0.002] |
|  | Not satisfied with their job | 0.122 | [0.003, 0.241] | 0.044 | 36.656 |  |  |

Supplementary Table 14: ATTs of 1 ADL on employment outcomes depending on whether individuals had pressure in their job at t (sensitivity analysis)

| **Outcome** | **Work pressure (t-1)** | **Estimate** | **Confidence Interval** | **p value** | **RSE** | **Second-diff estimate** | **Confidence interval** |  |
| --- | --- | --- | --- | --- | --- | --- | --- | --- |
| ***Model 1 - Workforce participation (n=5,906)*** | | | | | | | | |
| Full time | No work pressure | 0.006 | [-0.037, 0.049] | 0.797 | 1.697 | 0.079 | [0.005, 0.153] |  |
|  | Work pressure | -0.074 | [-0.134, -0.013] | 0.017 | -22.052 |  |  |  |
| Not in paid work | No work pressure | -0.038 | [-0.085, 0.01] | 0.121 | -11.298 | -0.136 | [-0.216, -0.055] |  |
|  | Work pressure | 0.098 | [0.033, 0.163] | 0.003 | 29.432 |  |  |  |
| Part time | No work pressure | 0.032 | [-0.021, 0.085] | 0.235 | 9.601 | 0.057 | [-0.024, 0.137] |  |
|  | Work pressure | -0.025 | [-0.085, 0.036] | 0.428 | -7.38 |  |  |  |
| ***Model 2 - Job changes (n=5,906)*** | | | | | | | | |
| In paid work, no new job | No work pressure | 0.038 | [-0.022, 0.098] | 0.217 | 11.376 | 0.064 | [-0.031, 0.159] |  |
|  | Work pressure | -0.026 | [-0.1, 0.048] | 0.488 | -7.841 |  |  |  |
| Looking for new job | No work pressure | -0.012 | [-0.024, 0] | 0.049 | -3.664 | 0.009 | [-0.018, 0.036] |  |
|  | Work pressure | -0.021 | [-0.045, 0.003] | 0.082 | -6.329 |  |  |  |
| New job | No work pressure | 0.01 | [-0.036, 0.056] | 0.665 | 3.067 | 0.051 | [-0.016, 0.119] |  |
|  | Work pressure | -0.041 | [-0.09, 0.008] | 0.099 | -12.364 |  |  |  |
| Not in paid work | No work pressure | -0.036 | [-0.082, 0.01] | 0.129 | -10.779 | -0.124 | [-0.202, -0.046] |  |
|  | Work pressure | 0.088 | [0.026, 0.151] | 0.006 | 26.534 |  |  |  |

Supplementary Table 15: ATTs of 1 ADL on employment outcomes depending on whether individuals were satisfied with their job at t (sensitivity analysis)

| **Outcome** | **Low satisfaction**  **(t-1)** | **Estimate** | **Confidence Interval** | | **p value** | **RSE** | **Second-diff estimate** | **Confidence interval** |
| --- | --- | --- | --- | --- | --- | --- | --- | --- |
| ***Model 1 - Workforce participation (n=6,947)*** | | | |  |  |  |  |  |
| Full time | Satisfied with their job | -0.016 | [-0.053, 0.02] | | 0.377 | -4.943 | 0.132 | [0.062, 0.203] |
|  | Not satisfied with their job | -0.149 | [-0.223, -0.075] | | 0 | -44.596 |  |  |
| Not in paid work | Satisfied with their job | -0.004 | [-0.043, 0.036] | | 0.857 | -1.093 | -0.117 | [-0.212, -0.022] |
|  | Not satisfied with their job | 0.113 | [0.017, 0.209] | | 0.021 | 33.909 |  |  |
| Part time | Satisfied with their job | 0.02 | [-0.022, 0.062] | | 0.35 | 6.036 | -0.016 | [-0.105, 0.074] |
|  | Not satisfied with their job | 0.036 | [-0.053, 0.124] | | 0.431 | 10.687 |  |  |
| ***Model 2 - Job changes (n=6,947)*** | | | |  |  |  |  |  |
| In paid work, no new job | Satisfied with their job | 0.002 | [-0.046, 0.05] | | 0.934 | 0.614 | 0.081 | [-0.013, 0.174] |
|  | Not satisfied with their job | -0.079 | [-0.173, 0.016] | | 0.103 | -23.598 |  |  |
| Looking for new job | Satisfied with their job | -0.015 | [-0.027, -0.002] | | 0.019 | -4.362 | 0.002 | [-0.016, 0.021] |
|  | Not satisfied with their job | -0.017 | [-0.044, 0.01] | | 0.215 | -5.084 |  |  |
| New job | Satisfied with their job | 0.018 | [-0.017, 0.053] | | 0.312 | 5.448 | 0.037 | [-0.011, 0.084] |
|  | Not satisfied with their job | -0.018 | [-0.065, 0.028] | | 0.437 | -5.53 |  |  |
| Not in paid work | Satisfied with their job | -0.006 | [-0.044, 0.033] | | 0.772 | -1.701 | -0.12 | [-0.216, -0.024] |
|  | Not satisfied with their job | 0.114 | [0.017, 0.211] | | 0.021 | 34.212 |  |  |

Supplementary Table 16: ATTs of 1 IADL on employment outcomes depending on whether individuals had pressure in their job at t (sensitivity analysis)

| **Outcome** | **Work pressure**  **(t-1)** | **Estimate** | **Confidence Interval** | **p value** | **RSE** | **Second-diff estimate** | **Confidence interval** |
| --- | --- | --- | --- | --- | --- | --- | --- |
| ***Model 1 - Workforce participation (n=6,032)*** | | | | | | | |
| Full time | No work pressure | -0.002 | [-0.048, 0.044] | 0.939 | -0.537 | 0.03 | [-0.046, 0.105] |
|  | Work pressure | -0.031 | [-0.091, 0.028] | 0.3 | -9.403 |  |  |
| Not in paid work | No work pressure | -0.034 | [-0.084, 0.017] | 0.189 | -10.16 | -0.105 | [-0.182, -0.028] |
|  | Work pressure | 0.071 | [0.013, 0.129] | 0.017 | 21.293 |  |  |
| Part time | No work pressure | 0.036 | [-0.019, 0.091] | 0.203 | 10.698 | 0.075 | [-0.001, 0.152] |
|  | Work pressure | -0.04 | [-0.093, 0.014] | 0.145 | -11.89 |  |  |
| ***Model 2 - Job changes (n=6,032)*** | | | | | | | |
| In paid work, no new job | No work pressure | 0.004 | [-0.06, 0.067] | 0.912 | 1.066 | 0.054 | [-0.041, 0.149] |
|  | Work pressure | -0.05 | [-0.122, 0.021] | 0.165 | -15.113 |  |  |
| Looking for new job | No work pressure | 0 | [-0.02, 0.02] | 0.988 | 0.045 | -0.022 | [-0.063, 0.019] |
|  | Work pressure | 0.022 | [-0.013, 0.058] | 0.223 | 6.618 |  |  |
| New job | No work pressure | 0.035 | [-0.014, 0.084] | 0.161 | 10.511 | 0.074 | [0.006, 0.143] |
|  | Work pressure | -0.039 | [-0.087, 0.008] | 0.102 | -11.825 |  |  |
| Not in paid work | No work pressure | -0.039 | [-0.087, 0.01] | 0.117 | -11.622 | -0.106 | [-0.181, -0.032] |
|  | Work pressure | 0.068 | [0.011, 0.125] | 0.02 | 20.32 |  |  |

Supplementary Table 17: ATTs of 1 IADL on employment outcomes depending on whether individuals were satisfied with their job at t (sensitivity analysis)

| **Outcome** | **Low satisfaction (t-1)** | **Estimate** | **Confidence Interval** | **p value** | **RSE** | **Second-diff estimate** | **Confidence interval** |
| --- | --- | --- | --- | --- | --- | --- | --- |
| ***Model 1 - Workforce participation (n=6,827)*** | | |  |  |  |  |  |
| Full time | Satisfied with their job | -0.013 | [-0.051, 0.025] | 0.496 | -3.914 | 0.034 | [-0.036, 0.103] |
|  | Not satisfied with their job | -0.047 | [-0.119, 0.026] | 0.206 | -14.031 |  |  |
| Not in paid work | Satisfied with their job | -0.003 | [-0.043, 0.036] | 0.864 | -1.046 | -0.071 | [-0.146, 0.005] |
|  | Not satisfied with their job | 0.067 | [-0.009, 0.143] | 0.083 | 20.104 |  |  |
| Part time | Satisfied with their job | 0.017 | [-0.025, 0.058] | 0.434 | 4.961 | 0.037 | [-0.013, 0.086] |
|  | Not satisfied with their job | -0.02 | [-0.062, 0.022] | 0.343 | -6.073 |  |  |
| ***Model 2 - Job changes (n=6,827)*** | | |  |  |  |  |  |
| In paid work, no new job | Satisfied with their job | -0.023 | [-0.072, 0.027] | 0.369 | -6.787 | 0.08 | [-0.006, 0.166] |
|  | Not satisfied with their job | -0.103 | [-0.19, -0.016] | 0.021 | -30.836 |  |  |
| Looking for new job | Satisfied with their job | 0.005 | [-0.013, 0.023] | 0.601 | 1.463 | -0.05 | [-0.12, 0.019] |
|  | Not satisfied with their job | 0.055 | [-0.017, 0.127] | 0.135 | 16.554 |  |  |
| New job | Satisfied with their job | 0.025 | [-0.011, 0.061] | 0.18 | 7.467 | 0.034 | [-0.014, 0.082] |
|  | Not satisfied with their job | -0.009 | [-0.055, 0.037] | 0.7 | -2.701 |  |  |
| Not in paid work | Satisfied with their job | -0.007 | [-0.046, 0.031] | 0.716 | -2.143 | -0.064 | [-0.138, 0.011] |
|  | Not satisfied with their job | 0.057 | [-0.019, 0.132] | 0.141 | 16.983 |  |  |

Supplementary Table 18: ATTs of a diagnosis (stroke, cancer, heart attack) on employment outcomes depending on whether individuals had pressure in their job at t (sensitivity analysis)

| **Outcome** | **Work pressure (t-1)** | **Estimate** | **Confidence Interval** | **p.value** | **RSE** | **Second-diff estimate** | **Confidence interval** |  |
| --- | --- | --- | --- | --- | --- | --- | --- | --- |
| ***Model 1 - Workforce participation (n=5,626)*** | | | | | | | | |
| Full time | No work pressure | -0.005 | [-0.056, 0.045] | 0.836 | -1.605 | 0.012 | [-0.07, 0.094] |  |
|  | Work pressure | -0.017 | [-0.082, 0.047] | 0.598 | -5.232 |  |  |  |
| Not in paid work | No work pressure | 0.019 | [-0.042, 0.08] | 0.544 | 5.698 | 0.022 | [-0.064, 0.107] |  |
|  | Work pressure | -0.003 | [-0.062, 0.057] | 0.926 | -0.848 |  |  |  |
| Part time | No work pressure | -0.014 | [-0.078, 0.051] | 0.679 | -4.093 | -0.034 | [-0.125, 0.058] |  |
|  | Work pressure | 0.02 | [-0.045, 0.085] | 0.54 | 6.079 |  |  |  |
| ***Model 2 - Job changes (n=5,626)*** | | | | | | | | |
| In paid work, no new job | No work pressure | -0.084 | [-0.156, -0.013] | 0.021 | -25.327 | -0.105 | [-0.21, 0] |  |
|  | Work pressure | 0.021 | [-0.056, 0.098] | 0.599 | 6.193 |  |  |  |
| Looking for new job | No work pressure | 0.015 | [-0.01, 0.041] | 0.245 | 4.561 | 0.034 | [-0.001, 0.069] |  |
|  | Work pressure | -0.019 | [-0.042, 0.005] | 0.118 | -5.636 |  |  |  |
| New job | No work pressure | 0.046 | [-0.007, 0.098] | 0.09 | 13.699 | 0.046 | [-0.033, 0.124] |  |
|  | Work pressure | 0 | [-0.058, 0.058] | 0.997 | 0.035 |  |  |  |
| Not in paid work | No work pressure | 0.024 | [-0.037, 0.084] | 0.444 | 7.067 | 0.026 | [-0.059, 0.11] |  |
|  | Work pressure | -0.002 | [-0.061, 0.057] | 0.948 | -0.593 |  |  |  |

Supplementary Table 19: ATTs of a diagnosis (stroke, cancer, heart attack) on employment outcomes depending on whether individuals were satisfied with their job at t (sensitivity analysis)

| **Outcome** | **Low satisfaction (t-1)** | **Estimate** | **Confidence Interval** | **p.value** | **RSE** | **Second-diff estimate** | **Confidence interval** |
| --- | --- | --- | --- | --- | --- | --- | --- |
| ***Model 1 - Workforce participation (n=6,308)*** | | |  |  |  |  |  |
| Full time | Satisfied with their job | 0.006 | [-0.034, 0.047] | 0.761 | 1.9 | 0.027 | [-0.046, 0.1] |
|  | Not satisfied with their job | -0.02 | [-0.095, 0.055] | 0.594 | -6.114 |  |  |
| Not in paid work | Satisfied with their job | 0.008 | [-0.037, 0.054] | 0.722 | 2.48 | -0.049 | [-0.14, 0.042] |
|  | Not satisfied with their job | 0.058 | [-0.033, 0.149] | 0.215 | 17.274 |  |  |
| Part time | Satisfied with their job | -0.015 | [-0.062, 0.033] | 0.55 | -4.38 | 0.023 | [-0.058, 0.103] |
|  | Not satisfied with their job | -0.037 | [-0.118, 0.044] | 0.369 | -11.16 |  |  |
| ***Model 2 - Job changes (n=6,308)*** | | |  |  |  |  |  |
| In paid work, no new job | Satisfied with their job | -0.028 | [-0.083, 0.027] | 0.314 | -8.468 | 0.122 | [0.026, 0.219] |
|  | Not satisfied with their job | -0.151 | [-0.249, -0.052] | 0.003 | -45.187 |  |  |
| Looking for new job | Satisfied with their job | -0.005 | [-0.019, 0.009] | 0.486 | -1.462 | -0.04 | [-0.115, 0.034] |
|  | Not satisfied with their job | 0.035 | [-0.044, 0.115] | 0.38 | 10.641 |  |  |
| New job | Satisfied with their job | 0.022 | [-0.018, 0.062] | 0.286 | 6.517 | -0.034 | [-0.127, 0.058] |
|  | Not satisfied with their job | 0.056 | [-0.038, 0.15] | 0.242 | 16.814 |  |  |
| Not in paid work | Satisfied with their job | 0.011 | [-0.034, 0.056] | 0.62 | 3.414 | -0.048 | [-0.14, 0.044] |
|  | Not satisfied with their job | 0.059 | [-0.033, 0.151] | 0.21 | 17.732 |  |  |

Supplementary Table 20: ATTs on labour market outcomes at time t+1 (individuals who did not experience an IADL or ADL onset between t and t+1 - sensitivity analysis)

| **Outcome** | **Estimate** | **Confidence interval** | **p value** | **Relative size effect** |
| --- | --- | --- | --- | --- |
| ***Model 1 - Workforce participation (n=10,497)*** | | |  |  |
| Full time | -0.01 | [-0.04, 0.01] | 0.28 | -4.2 |
| Not in paid work | 0.01 | [-0.01, 0.04] | 0.32 | 3.6 |
| Part time | 0 | [-0.02, 0.03] | 0.88 | 0.6 |
| ***Model 2 - Job changes (n=10,822)*** | |  |  |  |
| In paid work, no new job | -0.01 | [-0.04, 0.02] | 0.45 | -4.94 |
| Looking for new job | 0 | [-0.01, 0.01] | 0.97 | -0.07 |
| New job | 0 | [-0.02, 0.03] | 0.92 | 0.5 |
| Not in paid work | 0.01 | [-0.01, 0.03] | 0.34 | 4.52 |

Supplementary Table 21: ATTs on labour market outcomes at time t+1 depending on whether individuals had pressure in their job at t (individuals who did not experience an IADL or ADL onset between t and t+1 – sensitivity analysis)

| **Outcome** | **Work pressure (t-1)** | **Estimate** | **Confidence Interval** | **p.value** | **RSE** | **Second-diff estimate** | **Confidence interval** |
| --- | --- | --- | --- | --- | --- | --- | --- |
| ***Model 1 - Workforce participation (n=6,677)*** | | |  |  |  |  |  |
| Full time | No work pressure | 0.01 | [-0.03, 0.05] | 0.55 | 3.35 | 0.03 | [-0.03, 0.09] |
|  | Work pressure | -0.02 | [-0.07, 0.03] | 0.46 | -5.49 |  |  |
| Not in paid work | No work pressure | -0.04 | [-0.08, 0] | 0.06 | -11.47 | -0.09 | [-0.16, -0.03] |
|  | Work pressure | 0.06 | [0.01, 0.11] | 0.03 | 16.75 |  |  |
| Part time | No work pressure | 0.03 | [-0.02, 0.07] | 0.23 | 8.12 | 0.06 | [0, 0.13] |
|  | Work pressure | -0.04 | [-0.08, 0.01] | 0.11 | -11.26 |  |  |
| ***Model 2 - Job changes (n=6,677)*** | | |  |  |  |  |  |
| In paid work, no new job | No work pressure | 0.03 | [-0.02, 0.08] | 0.3 | 8.11 | 0.04 | [-0.04, 0.12] |
|  | Work pressure | -0.02 | [-0.08, 0.05] | 0.62 | -4.6 |  |  |
| Looking for new job | No work pressure | 0 | [-0.01, 0.01] | 0.97 | -0.09 | 0 | [-0.04, 0.03] |
|  | Work pressure | 0 | [-0.02, 0.03] | 0.74 | 1.35 |  |  |
| New job | No work pressure | 0.01 | [-0.03, 0.05] | 0.55 | 3.56 | 0.05 | [0, 0.11] |
|  | Work pressure | -0.04 | [-0.08, 0] | 0.05 | -12.35 |  |  |
| Not in paid work | No work pressure | -0.04 | [-0.08, 0] | 0.05 | -11.58 | -0.09 | [-0.15, -0.03] |
|  | Work pressure | 0.05 | [0, 0.1] | 0.04 | 15.59 |  |  |

Supplementary Table 22: ATTs on labour market outcomes at time t+1 depending on whether individuals were satisfied with their job at t (individuals who did not experience an IADL or ADL onset between t and t+1 - sensitivity analysis)

| **Outcome** | **Low satisfaction (t-1)** | **Estimate** | **Confidence Interval** | | **p.value** | | **RSE** | **Second-diff estimate** | **Confidence interval** |
| --- | --- | --- | --- | --- | --- | --- | --- | --- | --- |
| ***Model 1 - Workforce participation (n=7,122)*** | | | |  | |  |  |  |  |
| Full time | Satisfied with their job | 0 | [-0.03, 0.03] | | 0.97 | | 0.19 | 0.05 | [-0.01, 0.12] |
|  | Not satisfied with their job | -0.05 | [-0.12, 0.02] | | 0.13 | | -15.29 |  |  |
| Not in paid work | Satisfied with their job | -0.01 | [-0.04, 0.02] | | 0.61 | | -2.51 | -0.08 | [-0.15, -0.01] |
|  | Not satisfied with their job | 0.07 | [0, 0.14] | | 0.05 | | 21.51 |  |  |
| Part time | Satisfied with their job | 0.01 | [-0.03, 0.04] | | 0.66 | | 2.32 | 0.03 | [-0.03, 0.08] |
|  | Not satisfied with their job | -0.02 | [-0.07, 0.03] | | 0.42 | | -6.22 |  |  |
| ***Model 2 - Job changes (n=7,122)*** | | | |  | |  |  |  |  |
| In paid work, no new job | Satisfied with their job | 0 | [-0.04, 0.04] | | 0.93 | | -0.52 | 0.07 | [-0.01, 0.15] |
|  | Not satisfied with their job | -0.07 | [-0.15, 0.01] | | 0.08 | | -21.19 |  |  |
| Looking for new job | Satisfied with their job | 0 | [-0.01, 0.01] | | 0.86 | | -0.38 | -0.03 | [-0.08, 0.03] |
|  | Not satisfied with their job | 0.03 | [-0.03, 0.08] | | 0.35 | | 7.9 |  |  |
| New job | Satisfied with their job | 0.01 | [-0.02, 0.04] | | 0.34 | | 4.31 | 0.03 | [0, 0.07] |
|  | Not satisfied with their job | -0.02 | [-0.06, 0.02] | | 0.3 | | -5.91 |  |  |
| Not in paid work | Satisfied with their job | -0.01 | [-0.04, 0.02] | | 0.48 | | -3.41 | -0.08 | [-0.15, 0] |
|  | Not satisfied with their job | 0.06 | [-0.01, 0.14] | | 0.09 | | 19.2 |  |  |

Supplementary Table 23: Difference in the Average Marginal Effect (AME) of treated individuals between those whose partner is not employed versus those whose partner is still employed after the onset of their ADL or IADL limitation

| **Outcome** | **Contrast** | **Estimate** | **Confidence Interval** | **p-value** |
| --- | --- | --- | --- | --- |
| ***Model 1 - Workforce participation (n=11,248)*** | | | | |
| Full-time employed | Partner not employed - Partner employed | -0.07 | [-0.13, -0.02] | 0.009 |
| Not in paid work | Partner not employed - Partner employed | 0.13 | [0.07, 0.19] | <0.001 |
| Part-time employed | Partner not employed - Partner employed | -0.06 | [-0.11, 0.00] | 0.053 |
| ***Model 2 - Job changes (n=11,588)*** | | | | |
| In paid work, no new job | Partner not employed - Partner employed | -0.09 | [-0.16, -0.02] | 0.010 |
| Looking for new job | Partner not employed - Partner employed | -0.00 | [-0.03, 0.02] | 0.801 |
| New job | Partner not employed - Partner employed | -0.03 | [-0.08, 0.02] | 0.298 |
| Not in paid work | Partner not employed - Partner employed | 0.12 | [0.07, 0.18] | <0.001 |
